# Semi-automated approaches to optimize deep brain stimulation parameters in Parkinson’s disease

**DOI:** 10.1186/s12984-021-00873-9

**Published:** 2021-05-21

**Authors:** Kenneth H. Louie, Matthew N. Petrucci, Logan L. Grado, Chiahao Lu, Paul J. Tuite, Andrew G. Lamperski, Colum D. MacKinnon, Scott E. Cooper, Theoden I. Netoff

**Affiliations:** 1 Department of Biomedical Engineering, University of Minnesota, 312 Church St. SE, Minneapolis, MN 55455 US; 2 Department of Neurology, University of Minnesota, 516 Delaware St. SE, 55455 Minneapoli, MN US; 3 Department of Electrical and Computer Engineering, University of Minnesota, 200 Union St. SE, Minneapolis, MN 55455 US

**Keywords:** Bayesian optimization, Deep brain stimulation, Gaussian process, Probit, Parkinson’s disease, Rigidity

## Abstract

**Background:**

Deep brain stimulation (DBS) is a treatment option for Parkinson’s disease patients when medication does not sufficiently manage their symptoms. DBS can be a highly effect therapy, but only after a time-consuming trial-and-error stimulation parameter adjustment process that is susceptible to clinician bias. This trial-and-error process will be further prolonged with the introduction of segmented electrodes that are now commercially available. New approaches to optimizing a patient’s stimulation parameters, that can also handle the increasing complexity of new electrode and stimulator designs, is needed.

**Methods:**

To improve DBS parameter programming, we explored two semi-automated optimization approaches: a Bayesian optimization (BayesOpt) algorithm to efficiently determine a patient’s optimal stimulation parameter for minimizing rigidity, and a probit Gaussian process (pGP) to assess patient’s preference. Quantified rigidity measurements were obtained using a robotic manipulandum in two participants over two visits. Rigidity was measured, in 5Hz increments, between 10–185Hz (total 30–36 frequencies) on the first visit and at eight BayesOpt algorithm-selected frequencies on the second visit. The participant was also asked their preference between the current and previous stimulation frequency. First, we compared the optimal frequency between visits with the participant’s preferred frequency. Next, we evaluated the efficiency of the BayesOpt algorithm, comparing it to random and equal interval selection of frequency.

**Results:**

The BayesOpt algorithm estimated the optimal frequency to be the highest tolerable frequency, matching the optimal frequency found during the first visit. However, the participants’ pGP models indicate a preference at frequencies between 70–110 Hz. Here the stimulation frequency is lowest that achieves nearly maximal suppression of rigidity. BayesOpt was efficient, estimating the rigidity response curve to stimulation that was almost indistinguishable when compared to the longer brute force method.

**Conclusions:**

These results provide preliminary evidence of the feasibility to use BayesOpt for determining the optimal frequency, while pGP patient’s preferences include more difficult to measure outcomes. Both novel approaches can shorten DBS programming and can be expanded to include multiple symptoms and parameters.

## Introduction

Deep brain stimulation (DBS) is a highly effective therapeutic option for people with Parkinson’s disease (PD) [1–6]. However, efficacy of stimulation is dependent upon the stimulation waveform delivered. Three parameters describe the waveform: amplitude (voltage or current), frequency, and pulse width. These parameters have a wide range of possible values and are determined for each patient, individually, after a prolonged clinical optimization phase following implantation of electrodes in the subthalamic nucleus or globus pallidus interna. Current generation of DBS electrodes have multiple contacts (4 to 8), which the clinician will have to determine the best configuration (anodes and cathodes) as well. Given the range of possible waveform parameters and different electrode configuration, millions of possible setting combinations are possible. From a clinical perspective, the effective parameter space is much smaller. Many settings do not reach clinical effectiveness, have similar motor sign improvement, or are likely to induce side-effects and thus can be excluded from exploration [7, 8]. Nonetheless, the determination of “optimal” stimulation settings requires the clinician to iteratively modify stimulation parameters to best alleviate the individual’s motor signs. This can require 50 or more hours of parameter tuning [9]. Following this process, improvements in Unified Parkinson’s Disease Rating Scale (UPDRS) motor scores between 20-50% are often seen in individuals [2, 6, 10]. While the clinician optimization parameter improve UPDRS scores, it is still uncertain whether the clinician has identified the best stimulation settings using this tuning approach given that a large proportion of the parameter space remains untested.

Selecting the optimum stimulation settings state is further limited by the subjectivity and poor resolution of the motor outcomes measures. The clinical standard to evaluate the efficacy of stimulation is through examination of motor signs such as rigidity, bradykinesia, tremor, and gait. The severity of these motor signs is assessed using the motor UPDRS, which assigns a score to each element of the exam with an ordinal value between 0–4. This method of assessment and ordinal scoring limits the accuracy of outcome measurement, because it is subjective and limited in scope. Methods have been developed for the quantitative assessment of rigidity [11–14], bradykinesia [15–21], tremor [22], and gait [23–27], but these are rarely used to determine DBS settings [28, 29].

Several methods to increase the efficiency of DBS programming have previously been proposed. These include algorithms to select stimulation parameters based on iterative clinical assessments of benefits and side effects [7, 8, 30–32]. Yet, the parameter space tested with these approaches is still limited, and it is unclear if the greatest efficacy for a given motor sign has been achieved. Others have attempted to use biophysical models to determine settings that create a presumed ideal electrical field within a region of interest [33–37]. These electrical fields are modeled to selectively activate the desired brain tissue and minimize current spread to structures that may cause side effects. However, these techniques rely on high-resolution imaging of the lead and brain. This approach also assumes that one particular anatomical stimulation target is the most efficacious. Yet, there is currently a lack of consensus on the best target region(s) [38] or neural elements for symptomatic improvement. Finally, the most effective site for one symptom may be different from the best site for another [39–41], and the best target may differ between patients.

We posit that the determination of DBS settings can be substantially improved by using quantitative measures of motor signs obtained during standardized and controlled tasks. This approach increases the resolution of the outcome measure and removes potential bias.

Here we propose a semi-automated Bayesian optimization approach to tune stimulation parameters using quantitative real-time measures of motor function to discover patient specific optimized settings. Additionally, we modeled the patient preference to stimulation parameters using a semi-automated probit Gaussian Process based on pairwise comparisons. The Bayesian optimization approach used here differs from past optimization approaches as it creates a model of motor function response to stimulation parameters. This method of optimization combined with a standardized and controlled task to quantify motor function will provide a more efficient method to consistently model a patient’s motor function response to parameter adjustment. Therefore this method can consistently converge on the most efficacious stimulation settings. Fast convergence could minimize time and cost associated with determining optimal stimulation parameters, and this could also reduce the confounding effects of patient fatigue. Fatigue is known to significantly alter motor function. Consistent outcomes potentially reduce bias from the clinician, reducing the variance in UPDRS motor score improvements. Furthermore, Bayesian optimization can also help optimally resample parameters of interest to improve accuracy as well ensuring that the parameter space is adequately tested so that potentially good therapeutic settings are not missed. The BayesOpt and probit Gaussian Process approaches are described as semi-automated, as it depends on a clinician present to set parameters and to observe potential side effects. This study demonstrates the process and the feasibility of using a Bayesian optimization approach in two patients.

For the purposes of proof-of-principle, we chose to optimize stimulation frequency using real-time measures of forearm rigidity, obtained from a robotic manipulandum [42]. Rigidity was chosen as the motor outcome based on evidence that it rapidly responds to DBS [14], thus providing a reasonable wash-in period to rapidly assess the effects of stimulation frequency. Additionally, rigidity response to frequency has not been as widely studied as bradykinesia and tremor, and may not be monotonic. Lower frequencies have shown to yield little to no effect, or may even worsen rigidity. Conversely, stimulation at higher frequencies (> 60Hz) can produce a rapid and marked reduction in rigidity [14, 43].

There are two goals of this project, (1) develop a method for rapid and efficient semi-automated optimization of frequency to minimize rigidity, and (2) develop a method to determine an individual’s preferred stimulation frequency. A comparison between approaches will determine if the most efficacious settings differs from the patient’s preference. While the study only looked at one motor sign and stimulation parameter, the benefits of optimization using the Bayesian approach is expected to be greater when generalized to more than one parameter.

## Methods

### Participant demographics

Two individuals with PD (Table 1) were tested, one with DBS targeting the internal segment of the globus pallidus and the other with DBS targeting the subthalamic nucleus. Both individuals showed significant improvements in motor symptom severity (assessed using part III of the UPDRS) while on clinical stimulation settings compared to off stimulation (Participant 1: off DBS UPDRS III = 56, on DBS UPDRS III = 42; Participant 2: off DBS UPDRS III = 65, on DBS UPDRS III = 30). The protocol was approved by the local institutional review board and both individuals provided informed consent prior to participation. Testing was conducted in the off-medication state, with their last dose of Parkinson’s medication taken at least 16 h prior to their visit dates. Both participants were classified as having the akinetic-rigid subtype of PD based on the ratio between the participant’s UPDRS tremor and bradykinesia scores [44]. Throughout testing, DBS amplitude, pulse width, and electrode contacts settings were fixed to their clinically optimized settings.

**Table 1:** Participant demographics

Participant ID	Age	Sex	Disease duration (years)	Subtype	DBS target	UPDRS III rigidity (Side^a^)	Contacts	Amplitude	Pulse width	Clinical frequency	Optimized frequency
Participant 1	53	M	16	AR	GPi	2 (L)	1−2−c+	4.5 V	60$\mu$s	125Hz	155Hz
Participant 2	39	F	09	AR	STN	2 (R)	2−c+	2.7 V	60$\mu$s	150Hz	185Hz

Contacts, amplitude, pulse width, and frequency settings are taken from the contralateral side of testing

AR, akinetic-rigid

GPi, Globus Pallidus Interna, STN = Subthalamic Nucleus

^a^ Side of the body tested

### Rigidity measurement

**Fig. 1:**
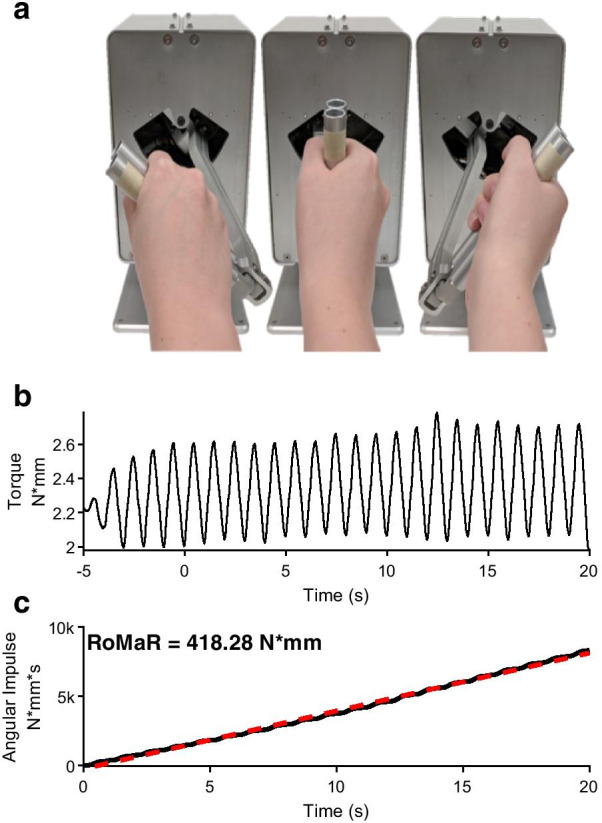
Rigidity quantification. **a** Robotic manipulandum. A participant is instructed to hold onto the handle as a motor moves it in a sinusoidal rhythm. Torque is measured through a strain gauge that is attached to the motor. **b** Estimating rigidity from torque data. Torque data is low pass filtered with a cutoff frequency of 20Hz and measured over a 20 s trial after a 5 s warm-up. **c** A patient’s RoMaR value is calculated by measuring the slope of the impulse of the filtered torque signal (red line)

A custom-made robotic manipulandum (Entact, Toronto, CA) was used to quantify rigidity. The robotic manipulandum houses a handle attached to a servomotor allowing rotation about the supination-pronation axis of the forearm (Fig. 1a). A strain gauge is attached to the shaft of the handle and is used to measure resistive torque. Torque is obtained from the strain gauge data after it is passed through a 16-bit digitizer (National Instrument, Austin, TX) sampling at 1KHz, followed by a digital low pass filter with a cutoff frequency of 20Hz using a 2 pole Butterworth filter (Fig. 1b). To estimate the force the participant imposes on the handle, the torque is rectified and integrated with respect to time, thus obtaining a measure of angular impulse [12]. The slope (vs. time) of the angular impulse was taken as the Robotic Manipulandum Rigidity (RoMaR) value, as shown in Fig. 1c. This method of quantifying rigidity has been validated with PD patient’s UPDRS Upper limb rigidity scores [42].

### Optimization

Considerable effort is needed by our PD participants to obtain multiple RoMaR values. Participants were asked to come into lab off PD medication and stimulation turned off prior to the experiment. Furthermore, motor signs are not sufficiently alleviated during the experiment, making it difficult for the participant to reach the end of the experiment. Considering these issues, not all optimization algorithms are viable. An efficient and cost-effective algorithm is needed. In this study we used a semi-automated Bayesian optimization approach to sample the participant’s specific rigidity-frequency relationship, as it has been shown to be efficient in terms of number of samples needed and useful when measurements are costly to obtain [45–49].

Our objective function for the semi-automated Bayesian optimization algorithm only includes a quantitative measure of rigidity. However, motor sign improvement *and* side effects are considered during clinical optimization. To include side effects into Bayesian optimization, a quantitative measure is needed. Side effects are difficult to quantify accurately, and rather than incorporating a coarse measurement into the objective function, a comparison approach can be taken. In conjunction with Bayesian optimization, subjects are asked to select their preferred setting when comparing their current setting to their previous setting. These pairwise comparisons are used to create a preference model at the end of the experiment using a probit Gaussian process.

#### Bayesian optimization

Bayesian optimization’s (BayesOpt’s) efficiency in sampling arises from incorporating prior beliefs about the input space with observations to build a model using Bayes’ theorem,

$$\begin{aligned} P(M \mid E) \propto P(E \mid M)P(M). \end{aligned}$$

This equation states that the *posterior* probability of the model *M*, given observations, *E*, is proportional to the likelihood of observing *E* given the model, multiplied by the *prior* probability of *M*. Once the model *M* has been built, BayesOpt operates on the model to direct sampling where it is beneficial to the goals of the optimization. Four steps are needed to run BayesOpt: (1) construction of a model from evidence, (2) estimate the mean and standard deviation at each frequency, (3) determine the utility of sampling at various frequencies, and 4) sample where utility is highest. These steps are repeated until some metric of model convergence is achieved.

Various methods can be used to create a model from evidence, but a Gaussian processes (GP) is preferred because it meets many “simple and natural” conditions common in many optimization tasks [45]:

$$\begin{aligned} f(x) \sim \mathcal {GP}(m(x),k(x,x')). \end{aligned}$$

A GP is a generalization of the Gaussian probability distribution and can be thought as a *distribution over functions* [50]. The GP is completely specified by its mean function, *m*(*x*), and covariance function, $k(x,x')$, which measures the “similarity” between any two frequencies *x* and $x'$. The mean function is often set to 0, but was set to the mean RoMaR value after testing four frequencies in our study. For the covariance function, we used the Matérn kernel [51, 52] as it offers flexibility between the smoothness of the input-output response and is among the most common kernels for GPs [49]:

$$\begin{aligned} k&(x_i,x_j) = \\ &\frac{1}{2^{\nu-1}\Gamma(\nu)}(2\sqrt \nu \lVert x_i - x_j \rVert)^\nu H_\nu (2\sqrt \nu \lVert x_i - x_j \rVert), \end{aligned}$$

where $\Gamma (\cdot )$ is the Gamma function, and $H_\nu$ is the Bessel function of order $\nu$. Smoothness of the covariance function is balanced through the order parameter, $\nu$, and the length-constant $\ell$. For this study, $\nu =3/2$ as we expected to observe coarseness in the data but a smooth trend, and estimated $\ell$ using MATLAB’s hyperparameter optimization (MATLAB 2017b, Mathworks Inc., Natick, MA) for each participant.

Now that we have built a model from our evidence, we can estimate the mean and standard deviation of RoMaR values at each frequency; this estimation is also called the *predictive* distribution. To calculate the *predictive* distribution, we incorporate all of our observations, $\mathcal {D}_{1:n} = {x_i, f(x_i)}$, with our GP model. Let us define a vector containing all values $\mathbf{f} _{1:n} = [f(x_1),...,f(x_n)]$. An expression for the *predictive* distribution can be derived as,

$$\begin{aligned} P(f_{n+1} \mid \mathcal {D}_{1:n}, x_{n+1}) = \mathcal {N}(\mu _n(x_{n+1},\sigma _{n+1}^2(x_{n+1})) \end{aligned}$$

where

$$\begin{aligned} \mu _n(x_{n+1})= & {} \mathbf {k^T}\mathbf {K}^{-1}\mathbf {f}_{1:n},\\ \sigma _n^2(x_{n+1})= & {} k(x_{n+1},x_{n+1}) - \mathbf {k^T}\mathbf {K}^{-1}\mathbf {k}. \end{aligned}$$

We denote $\mathbf {k}$ as a vector of covariance $[k(x_{n+1},x_1),...,k(x_{n+1},x_n)]$, and $\mathbf {K}$ as a matrix containing all covariance values where the *i*, *j* entry is the covariance $k(x_i,x_j)$.

We use the information contained in the *predictive* distribution to determine where to sample next. Using the mean and standard deviation, BayesOpt selects the frequency where the utility, *u*(*x*), is greatest. In this study, utility was defined as the expected improvement to the model’s estimated minimum at each frequency:

$$\begin{aligned} \small u(x)= & {} {\left\{ \begin{array}{ll} (\mu (x) - f(x^-))\Phi (Z) + \sigma (x)\phi (Z) &{} if \sigma (x) > 0 \\ 0 &{} if \sigma (x) = 0 \end{array}\right. }\\ Z= & {} \frac{\mu (x) - f(x^-)}{\sigma (x)} \end{aligned}$$

where $x^- = argmin_{x_i \in x_{1:n}}f(x_i)$, $\Phi (\cdot )$ is the cumulative density function of a standard normal distribution, and $\phi (\cdot )$ is the probability density function of a standard normal distribution. Sampling only where it is expected to improve the greatest can oversample an area of the input space, and choose a local minimum as the optimized frequency. MATLAB provides a method to account for oversampling through an exploration ratio that balances exploration and sampling near the estimated minimum. We use the MATLAB’s “bayesopt” function to calculate the utility at all frequencies using MATLAB’s expected improvement function and set an exploration ratio of 0.5. MATLAB returns the frequency with the largest utility to sample:

$$\begin{aligned} x_{n+1} = argmax_{x_{n+1}}u(x_{n+1} \mid \mathcal {D}_{1:n}). \end{aligned}$$

Sampling where the variance is greatest will provide new evidence and reduce the model uncertainty at that setting; improving the GP model’s estimation of the underlying rigidity response. However, the magnitude of the frequency could induce intolerable side effects in human participants. To safely test the frequency with the highest utility, a clinician was tasked with changing the frequency and monitor the participant for side effects. New evidence from sampling was used to update the GP model. See below for more information regarding the experimental protocol.

#### Probit gaussian process

An optimization algorithm depends on the metric used to measure the outcome. In previous sections, we have focused on optimizing rigidity, which ignores other symptom reductions and side effects caused by stimulation. The ultimate goal is not just to reduce rigidity, *but also to minimize side effects and improve overall quality of life.* Unfortunately, side effects and quality of life are difficult to measure, and to optimize for. Even if accurate rigidity and side effect measurements could be obtained during optimization, we would have to choose a function that balances the value of rigidity and side effects into a single scalar value.

Instead, we can re-formulate the problem and attempt to optimize directly for the desired outcome: *patient preference.* Asking patients to provide ratings on a numerical scale is difficult for the patients, often inaccurate, and subject to cognitive biases [53, 54]. However, humans excel at comparing options and expressing preference for one over the others [55]. In applications requiring human judgment, preference between two options is often more accurate than numerical ratings [56, 57].

In order to use binary preference data, we must reformulate the GP into a probit Gaussian process (pGP). In a pGP, instead of being able to directly sample the objective function, we must infer it from a set of binary observations. Our data is no longer a score at each setting, but is a set of ranked pairs $\mathcal {D} = \{a_i \succ b_i\}^m_{i=1}$, where $\succ$ indicates that a patient prefers setting *a* to *b*. We use $x_{1:m}$ to denote the *m* distinct comparisons in the training data, where $a_i$ and $b_i$ are two elements of $x_{1:m}$.

We model the value function $v(\cdot )$ for any setting as $v(\cdot ) = f(\cdot ) + \epsilon$, where $\epsilon \sim \mathcal {N}(0,\sigma ^2)$ is normally distributed noise. The value function describes the value a patient derives from a given setting, with the noise term modeling uncertainty in the participant’s preferences. Here, $\sigma$ is set to 1, and is not learned. The challenge here is to learn *f*, where $f = \{f(x_i)\}_{i=1}^n$ is the value of the objective function at the training points.

**Fig. 2:**
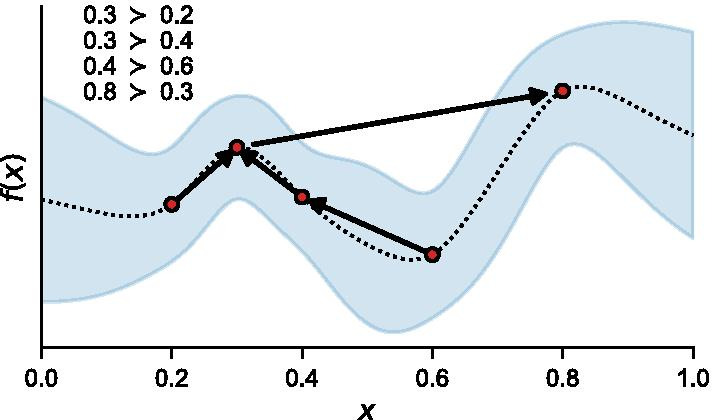
Example of the probit model inferring a GP from a set of preference data. Five evaluated inputs, *f(x)*, selected from a one-dimensional input space, *x*. Four comparisons are made between two evaluated inputs and are listed in the top left. Preference for an input is noted by being on the left side of the arrow, with the input it was compared to on the right of the arrow

We can now relate our binary observations back to the latent objective function using binomial-probit regression. An example of the probit procedure used to model a 1D function from a series of preferences is illustrated in Fig. 2. Using this model, the probability that item *a* is preferred to *b* can be calculated as:

$$\begin{aligned} P(a_i \succ b_i|f(a_i), f(b_i))&= P(v(a_i)> v(b_i)|f(a_i), f(b_i)) \\&= P(f(a_i) - f(b_i) > \epsilon _b - \epsilon _a) \\&= \Phi \Big (\frac{f(a_i)-f(b_i)}{\sqrt{2}\sigma }\Big ) \end{aligned}$$

where $\Phi (\cdot )$ is the CDF of the standard normal distribution. The probability that *a* is preferred to *b* is proportional to the difference between *f*(*a*) and *f*(*b*), divided by the magnitude of the noise, $\sigma$ [49].

Now, we can estimate the posterior distribution of the latent objective function. Our goal is to find the distribution of the objective function which maximizes the posterior probability of our binary observations, known as the *maximum a posterior* (MAP) estimate. We use Newton-Raphson recursion to learn $f_\text {MAP}$:

$$\begin{aligned} f_\text{MAP} = \mathrm{argmax}_{f} & P(f|\mathcal{D}) \propto \\ & P(f) \prod_{i=1}^{n}P(a_i \succ b_i | f(a_i),f(b_i)). \end{aligned}$$

Newton-Raphson recursion is subject to becoming stuck in local minima, so multiple restarts with random initial conditions ($N=25$) was performed.

After computing the model of the objective function, $f_\text {MAP}$, we can derive the predictive distribution of $P(f_{t+1}|f_\text {MAP},\mathcal {D})$:

$$\begin{aligned} P(f_{t+1}|f_\text{MAP}&,\mathcal{D}) \propto \mathcal{N} (\mathbf{k}\mathbf{K}^{-1} f_\text{MAP},\\ & k(x_{t+1},x_{t+1})-\mathbf{k}^T(\mathbf{K}+\mathbf{C}^{-1})^{-1}\mathbf{k}), \end{aligned}$$

where $\mathbf {C}$ is a matrix whose *m*, *n*th entry is given by

$$\begin{aligned} C_{m,n} = - \frac{\partial ^2}{\partial f(x_m) \partial f(x_n)}\sum ^{M}_{i=1} \log \Phi (Z_i) \end{aligned}$$

and

$$\begin{aligned} Z_i=\frac{f(a_i)-f(b_i)}{\sqrt{2}\sigma }. \end{aligned}$$

As before, $\mathbf {k}$ and $\mathbf {K}$ are the vector and matrix of covariances between inputs *x*.

Using the predictive distribution, we can then compute the utility and proceed with sampling as described in Bayesian Optimization.

### Experimental protocol

**Fig. 3:**
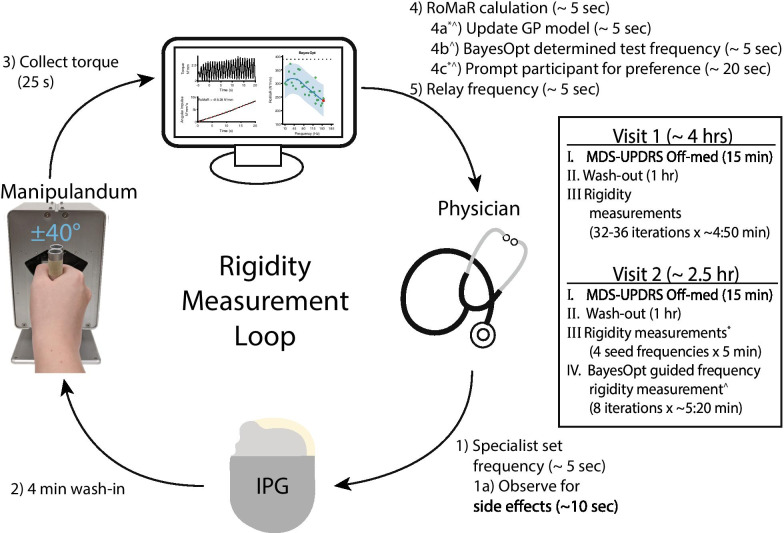
Loop schematic and experiment flow chart. Visit 1 and 2 both measure UPDRS and is followed by 1 h of stimulation washout. After washout, rigidity is measured at all frequencies between 10-185 Hz during visit 1, and at 4 seed frequencies (30, 80, 90, and 140 Hz) during visit 2. Visit 2 then uses BayesOpt to guide frequency selection for 8 iterations. All rigidity measurements follows the loop diagram starting with the movement disorder specialist programming the frequency on the participant’s implantable pulse generator

This study consisted of two visits. On the first visit, a brute-force approach was used to measure the RoMaR value at all frequencies, in pseudorandom order, between 10 and 185Hz in increments of 5Hz. Two additional measurements at the participant’s clinical frequency at the beginning and end of the experiment. Frequencies that produced uncomfortable side effects were halted and all higher frequencies were not tested. On the second visit, RoMaR values was obtained at four seed frequencies, 30, 80, 90, and 140 Hz prior to BayesOpt guided programming. These four frequencies were selected prior to the first participant and spanned a range that would be comfortable for most PD patients, with two samples (80Hz and 90Hz) near the anticipated transition between ineffective and suppressive stimulation frequencies that is seen in most patients. After testing at these seed frequencies, BayesOpt selected the next frequency to test for 8 iterations and incorporated frequency boundaries discovered during the first visit. One hour was allocated to testing, which included testing the 4 seed frequencies, data analysis and Bayesian optimization for setting selection, and the 8 tests, for a total of 12 rigidity measurements and 11 preference choices for each participant. The first rigidity measurement on each visit occurred after DBS has been turned off for 1 h. For both visits, frequencies were programmed into the implantable pulse generator by a movement disorder specialist who could observe any notable side effects and terminate the stimulation if necessary (Fig. 3).

Each rigidity measurement follows these steps: clinician sets stimulation frequency and monitors for side effects, 4 min of wash-in, custom software controlled 25 s of passive movement imposed by the manipulandum with a range of motion of $\pm \text {40}^\circ$, custom software calculation of the RoMaR value, and relay of the next frequency to the clinician. For visit 2 there are three additional steps: update of the GP model with the calculated RoMaR value, BayesOpt determined next frequency to test, and prompt the participant for frequency preference. Specifically, preference was asked with the following: “Which did you prefer, the current setting or the previous one, meaning the setting that felt best to you in terms of symptom reduction but also side effects?” The total time between each rigidity measurement takes was approximately 5 mins, with the majority of that time dedicated to wash-in time.

### Data analysis

A GP was to fit to the patient data sampled using the brute-force and the BayesOpt methods, and a pGP was created at the conclusion of the BayesOpt experiment. Two metrics are derived from the GP models: (1) the optimal frequency and (2) the frequency range considered indistinguishable from the minimum. The optimal frequency for rigidity suppression was estimated where the GP model’s mean was at a minimum. The frequency range was estimated as all frequencies whose rigidity scores fell within one standard deviation of the value at the minimum. We then compared the optimal frequency found between BayesOpt and the brute-force approach.

Next, to analyze the efficiency of the BayesOpt method we simulated two other sampling methods from the brute-force data and compared the frequency range of the estimated curves. One simulated approach was to sample between 10Hz and the maximum frequency each participant could tolerate in *approximately* equal intervals. For example, sampling at three points between 10 and the maximum frequency of Participant 1 (155 Hz), we would select brute-force RoMaR values at 10, 85, and 155 Hz. When the boundary of an interval was not one of the brute-force frequency tested, the frequency was rounded to the nearest tested setting. The second simulated approach randomly selected frequencies, with repeats allowed. Each sampling method was simulated with increasing number of included brute-force RoMaR values, fitting a GP at each iteration. Additionally, the random frequency selection was repeated 50 times. The frequency range of the GPs were stored for each method and compared to the BayesOpt method.

Lastly, we analyzed the peak of the participant’s stimulation frequency preference model to the optimal frequency of the BayesOpt GP model.

## Results

The objective of the study was to develop two methods: (1) a rapid and efficient semi-automated approach to optimize stimulation frequency to minimize rigidity, and (2) a method to determine an individual’s preferred stimulation frequency. We evaluate the efficiency of the semi-automated approach (BayesOpt) by comparing it to brute-force methods. Additionally, BayesOpts reliability was also investigated and compared to brute-force methods. Individual preferred stimulation frequency was compared to the results of BayesOpt. Two participants had two separate visits for optimization. In the first visit, the stimulation frequency was systematically tested at 30 frequencies for Participant 1 and 36 frequencies for Participant 2. For Participant 1, only stimulation frequencies at or below 155Hz were tolerable. In total, visit 1 (brute-force method) testing took  4 h. In the second visit (BayesOpt approach), the rigidity response curve to stimulation frequencies tested were selected during the study using the BayesOpt algorithm. During the BayesOpt experiment, we also asked patients to report which stimulation setting they preferred, the current stimulation frequency or the previous frequency tested, to estimate their preference for stimulation frequency. The pairwise comparisons were then quantified to provide a value of each setting using a pGP. Visit 2 testing (BayesOpt) took  2.5 h. Here we report four results: (1) how rigidity is dependent on stimulation frequency, (2) a comparison of rigidity’s dependency on frequency fit with GP models from the brute-force and the BayesOpt sampling, (3) evaluation of the efficiency in estimating the rigidity-frequency curve using BayesOpt, and (4) the participant’s preference for stimulation frequency, as estimated using the pGP.

**Fig. 4:**
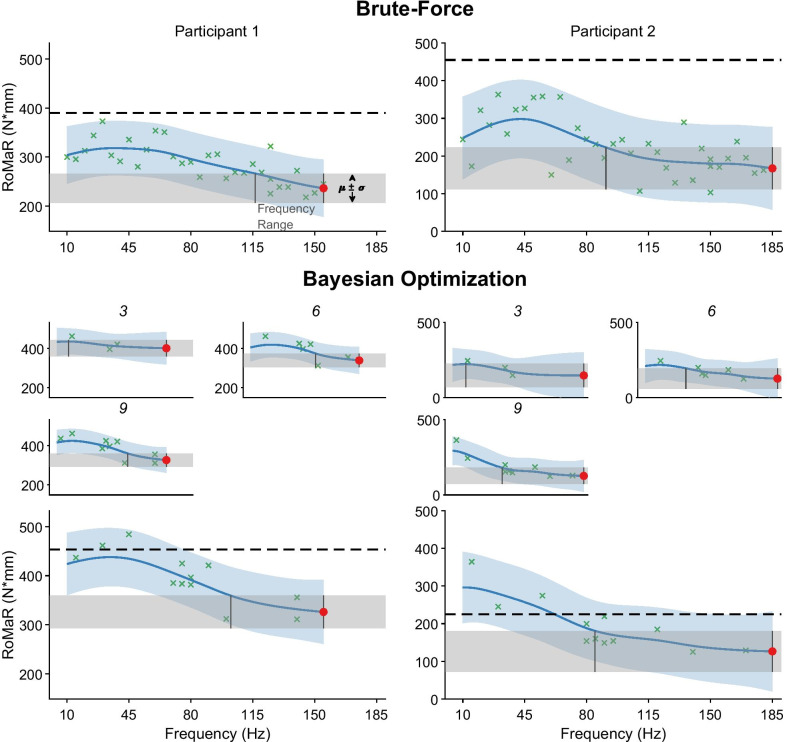
GP fits after Brute-force and BayesOpt optimization programming. Two PD participants (column) have had their rigidity response curve measured using two different sampling methods. Brute-force method tested all frequencies spaced by 5Hz from 10-185Hz, and sampled in pseudorandom order, top row. Bayesian optimization guided frequency testing for rigidity, bottom rows. Smaller plots shows the fitted GP model evaluated at 3, 6, and 9 frequencies selected by the BayesOpt method. The bottom row shows the final GP fitted model after 12 iterations of the BayesOpt method. *Black dashed lines* indicate the participant’s RoMaR value after 1 h off stimulation. *Red dot* indicates the estimated frequency that minimizes rigidity. *Shaded gray* region indicates RoMaR values within $\mu \pm \sigma$ of the optimal frequency and the frequency range is defined as all frequencies whose estimated RoMaR values fall within this range and is indicated by the two thin vertical lines

In both participants we observed that stimulation frequency greater than 80Hz was more effective at reducing rigidity than low frequency stimulation (Fig. 4, Brute-force). Interestingly, 10Hz in both participants reduces rigidity greater than 20–50Hz stimulation. Rigidity does not follow a steady decrease after 50Hz for Participant 2, where rigidity is higher between 130–155Hz than frequencies around it. Participant 1 does show a steady decrease after 50Hz, where their rigidity suppression was proportional to the stimulation frequency.

Rigidity was minimized at the highest frequency tolerated, or tested, at 155Hz for Participant 1 and185Hz for Participant 2 during their brute-force visit (Fig. 4, Brute-force *Red dot*). These stimulation frequencies were also estimated to minimize the participant’s rigidity from their BayesOpt model, which was determined using less than half of the number of evaluations needed for the brute-force model. The GP models between the two visits also had comparable accuracy, as evaluated by the model’s frequency range. For the brute-force model, the frequency range that suppressed rigidity was 39Hz wide for Participant 1 and 94Hz for Participant 2. BayesOpt models had frequency ranges that suppressed rigidity of 53Hz for Participant 1 and 100Hz for Participant 2, a difference of 14Hz and 6Hz.

**Fig. 5:**
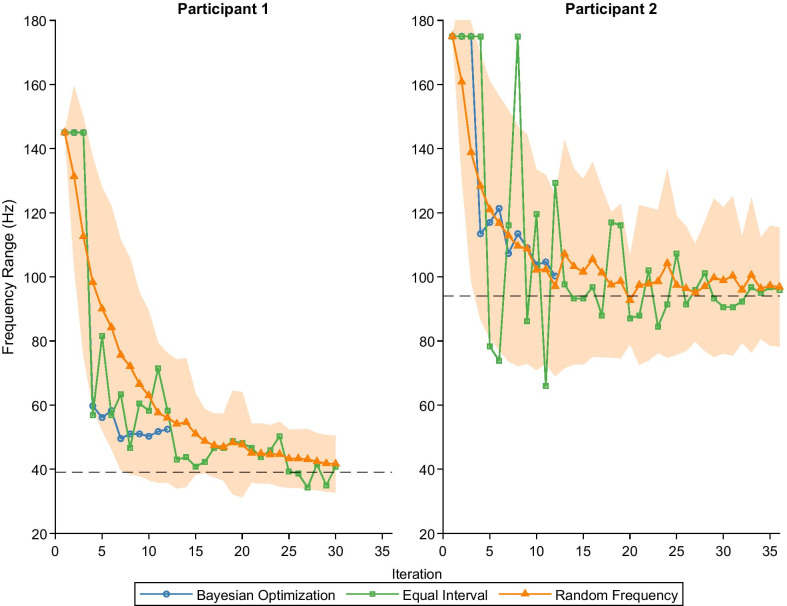
Efficiency comparison analysis of different methods. Frequency range (bottom) after each iteration of Bayesian optimization (*blue circles*) and two post-hoc frequency selection methods. The first post-hoc frequency selection method sampled approximately equal intervals 5Hz (*green squares*). The second method randomly selected frequencies (*orange triangles*), and the shaded light orange region indicates the standard deviation of 50 simulations at each iteration. *dashed line* indicates the frequency range of the full brute-force GP model. The frequency range measured the distance between the optimal frequency and the lowest frequency that is contained within the optimal frequency’s mean and standard deviation

Efficiency and reliability of the BayesOpt algorithm was evaluated through comparison between two different sampling methods: equal interval and random sampling of the frequency space (Fig. 5). The frequency range of rigidity suppression decreased for each sampling method as the iteration number increased for both participants. BayesOpt results in a large frequency range decrease after 4 iterations for both participants, followed by smaller increases or decreases as the number of iterations increases (*blue circles*). Equal interval sampling showed a similar performance as BayesOpt in Participant 1, but with larger changes in frequency range as the iteration number increased (*green circles*). In Participant 2, equal interval sampling achieved the smallest frequency range, but also had the largest variation in frequency range. Random sampling, on average, had the slowest decrease in frequency range of the different sampling methods (*orange triangle*). The standard deviation of random sampling decreased for Participant 1 as the iteration increased. However, the standard deviation did not change appreciably after 10 iterations for Participant 2.

**Fig. 6:**
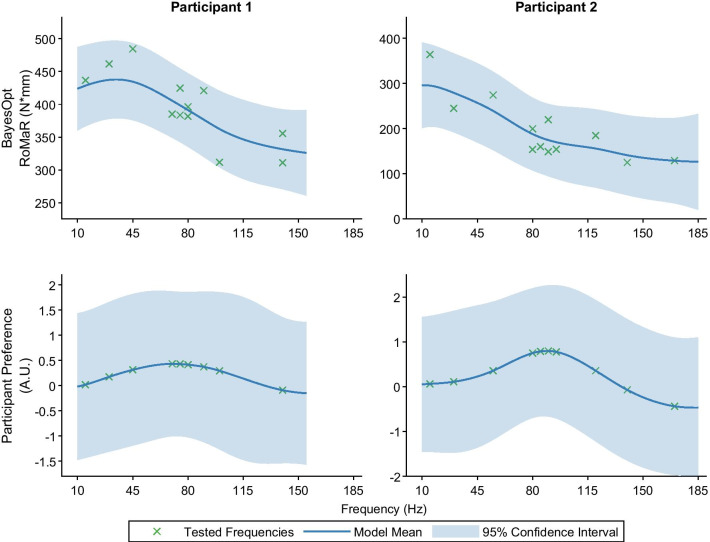
Probit Gaussian process fits. Rigidity response to frequency model after BayesOpt compared to preference model. The BayesOpt model was created after 12 iterations of the algorithm. The participants were asked which setting they preferred (the current or previous frequency setting). This was used to obtain their preference model. The average expected preference an input has over the entire input space is shown as the solid blue line, and the 95% confidence interval is shown as the shaded blue region

While testing different stimulation frequencies during Bayesian optimization, we asked patients to evaluate which setting they preferred: the current or previous setting. Based on the pairwise comparisons, the value of settings as a function of frequency was estimated using a probit Gaussian process. Fig. 6 shows the GP fit to the rigidity-frequency and pGP fit for preference-frequency for both participants. Both participants *did not* prefer frequencies that provided the minimum rigidity scores at the top of the stimulation frequency range, as one might expect. Instead, they preferred frequencies between 70–110Hz, the lowest frequencies that achieved around 80% of the benefit seen at the maximum rigidity suppression. Participant 1 strongest preference is at 70Hz, and Participant 2 at 89Hz.

## Discussion

Here we have demonstrated the feasibility of a semi-automated Bayesian optimization approach to rapidly and efficiently determine a single stimulation parameter (frequency) to minimize a single motor sign of Parkinson’s Disease. The approach was able to model the participant’s rigidity response efficiently and accurately. Additionally, we explored the difference between an individual’s preference and response model. By using Bayesian optimization, the optimal frequency for maximum reduced rigidity was determined from fewer sampled frequencies than those using the brute-force estimation method. Bayesian optimization also reduced the frequency range it sampled over quickly with small changes with increasing number of samples, improving the confidence that the optimal frequency was found. When compared to random sampling, equal interval sampling, and BayesOpt methods, BayesOpt was able to more efficiently and reliably estimate the rigidity’s sensitivity to frequency and narrow down the frequency range where the minimum rigidity is observed. Considering balancing the accuracy of the curve fit and narrowing the range of the frequency range around the minimum rigidity, this suggests that BayesOpt is the most successful sampling algorithm. When measuring patient preferences between different frequency settings, we found that their preferred stimulation frequency is not at the frequency where rigidity is most suppressed, but at the minimum frequency at which rigidity significantly suppressed.

The pGP models, constructed from the patients’ preference, revealed that both participants preferred stimulation frequencies that differed from the frequency that maximally reduced rigidity. The difference may be explained by the limitation of the RoMaR value to capture side effects of the stimulation. Side effects of DBS include motor and non-motor effects. Some motor side effects, such as dyskinesia, are easily detectable by an examiner, but more subtle or non-motor side effects like mood changes [58, 59] may be apparent only to the patient. Impairment of verbal fluency, induced at higher stimulation frequencies, [60] is another subtle symptom that is more easily detected by the patient. Our findings suggest that patients may prefer stimulation settings that balance effects of multiple symptoms and side effects. How patients weigh the significance and severity of multiple factors might differ among individuals and is an open question for future research.

We tested participants with a predominantly akinetic-rigid subtype of PD because we expected that rigidity in these individuals would have a strong dependence on stimulation parameters. Our findings may only be applicable to people with a similar phenotype. However, the efficacy of DBS on other motor signs, such as tremor and bradykinesia, is also dependent upon stimulation frequency [61, 62]. Accordingly, this semi-automated approach could be applied to optimize other motor signs that can be readily be quantified in real-time.

### Limitations

The data set used here is limited and the results may not generalize to other participants, but the optimization approach should. Participants in this study were classified as the akinetic-rigid subtype of PD and had disease durations of 9 and 16 years. Patients who are tremor dominant may not have rigidity that responds strongly to frequency. Additionally, patients that are in the early stages of PD may not present with RoMaR values greater than healthy older adults. However, the average disease duration prior to receiving DBS therapy is 13 years [63]. This duration is greater than Participant’s 2 disease duration. Also, while tremor dominant subtype patients may not benefit from this approach, our findings indicate that akinetic-rigid subtypes can benefit. Furthermore, our results also indicate that the BayesOpt approach can be effective regardless of stimulation target. Participant 1 had stimulation target in the internal segment of the globus pallidus, and Participant 2 had stimulation in the subthalamic nucleus. Both subjects showed a similar rigidity response to changes in frequency, which is in broad agreement with previous studies showing that the motor benefits of DBS are similar between the two targets [10, 64–68]. Given the results of the study, we expect similar outcomes in a larger group of akinetic-rigid PD subtypes for rigidity.

A potential confound in this study was the duration of time between visits. In Participants 1 and 2 the time between visits were 4.5 and 9 months respectively. These time periods are sufficiently long that expression of rigidity may have changed between tests. In a 5-year longitudinal study, Holden et al. found that clinical motor scores increased 2.4 points per year [69]. For Participant 1, an increase in the UPDRS III right arm rigidity scores increased by one ordinal point from visit 1 to 2, and this is reflected in the higher RoMaR values seen during visit 2. Participant 2, however, had no change in clinical rigidity score, and but their RoMaR values were lower during visit 2. This may reflect day-to-day fluctuations in motor signs and differences in medication wash-out between visits, which are not captured by the clinical rating scoring system. Nonetheless, despite changes in the mean in RoMaR values, the shape of the response curve was consistent for both the brute-force and the BayesOpt measured curves.

While this study focused on optimizing RoMaR values alone, total energy delivered (TEED) may also be considered [70] when optimizing stimulation parameters. However, it has also been observed that TEED may not play as critical of a role as the frequency when tuning stimulation parameters [71]. If one desires to add TEED to the optimization, TEED may be scaled and then added to the RoMaR value for a total cost. Alternatively, stimulation amplitude can be adjusted to compensate for changes in frequency to keep TEED the same across the study. In this study, we neither accounted for nor compensated for changes in TEED in the optimization. In a study by Rizzone et al. showed that lower frequencies required a larger stimulation amplitude than higher frequencies to reach clinical effectiveness [30]. Therefore, in our study, the TEED may have been below therapeutic levels with stimulation at low frequencies. However, both participants showed that 10Hz stimulation was more effective at reducing rigidity than 20–50 Hz (Fig. 4, top row). These results may indicate that the rigidity dependence on frequency is not monotonic, but this was not explored in detail. Keeping TEED constant may be beneficial when optimizing for one stimulation parameter but may introduce difficult trade-off considerations when optimizing over several parameters. For example, shortening the battery life to obtain the greatest reduction in motor symptoms.

Variability in RoMaR values influences the Gaussian process model and can influence the acquisition function within the BayesOpt algorithm. The acquisition function used in this study was the expected improvement function, which selects the next point to sample based on mean and variance. Unfortunately, we could not evaluate how rigidity variability influences the BayesOpt algorithm with our brute-force and BayesOpt data set. In the brute-force data set, time was limited given that the participants were in the off-medicate state and due to the amount of time allocated for new stimulation frequency settings to wash-in. In the BayesOpt data set, we did not control what frequencies to sample rigidity at. Some frequencies were tested more than once, but all frequencies are not guaranteed to be tested when the number of iterations of the BayesOpt algorithm is less than the number of all possible frequencies we could test.

Other optimization approaches may perform as well, or outperform, the BayesOpt algorithm when optimizing one stimulation setting to one motor symptom of Parkinson’s disease. When comparing the frequency range of the Gaussian process models as more data is included (Fig. 5), Equal Interval frequency sampling performed as well as the BayesOpt algorithm. One could predict that Equal Interval sampling with a faster increase in number of intervals could improve the frequency range faster than the BayesOpt algorithm. However, extending the Equal Interval method to more stimulation parameters and motor symptoms will quickly increase the amount of sampling that is needed. This will, in turn, increase the duration needed to optimization parameters. Although the BayesOpt algorithm did not outperform a simple brute-force approach in the one stimulation parameter one motor symptom case, we expect the efficiency of the BayesOpt algorithm to be more pronounced when additional parameters and symptoms are included in the optimization.

Both approaches in this study are semi-automated to show proof-of-principal, but the goal is to create a fully automated closed-loop algorithm that incorporates additional motor signs and stimulation parameters. To achieve a fully automated closed-loop BayesOpt algorithm, objective measures of side effects, a single metric of the tested parameter’s efficacy, and external software access to the patient’s implantable pulse generator are needed. Currently, no methods exist for automated side effect detection. A single metric of the parameter’s efficacy can be simple. For example, one could normalize all measures to the patient with their off-stimulation baseline, and simply sum the measures to create a single metric. More complex combinations of metrics are possible, such as adding weights to different motor sign measures. Direct programming of an implantable pulse generator from a computer is only available with Medtronic series of brain stimulators, currently limiting an automated approach, but in the future we expect more stimulators will be directly programmable.

### Future directions

A fully automated closed-loop algorithm may also be built off of patient preference data, rather than quantified metrics of motor scores. A patient could test settings and evaluate their preferences and BayesOpt can be used to suggest new settings to test. The advantage of this approach is that patient preference may account for side effects that may be difficult to measure quantitatively and include in a cost function for optimization.

As implantable stimulators get more complicated and clinicians are provided with more flexibility, the opportunity to create better patient specific settings will be offset by the time it takes to find those optimal parameters and the complexity of the search. Efficient tuning will be necessary, especially when motor signs with long wash-in and wash-out times are evaluated. For the motor signs of bradykinesia and tremor, effects of stimulation can be seen within a couple of minutes [72]. Since our protocol allows for 4 mins of wash-in time, both motor signs can be evaluated after this period with additional time (1 min) for each measurement. If measurements of gait are added, *1 hour* of wash-in time is needed before evaluation [72]. Approximately 30 steps are needed to measure gait, collected over 2 mins [73]. Using an approach like BayesOpt enables testing stimulation setting effects on such motor signs that otherwise would not be possible with other approaches given the constraints of a clinical visit and the endurance of a patient.

## Conclusion

In summary, this study provides proof-of-principle of setting selection using a semi-automated BayesOpts for tuning frequency to minimize rigidity scores and pGP for evaluating patient preferences. BayesOpts provides a rigorous approach to efficiently and accurately determining the optimal frequency to reduce rigidity, and pGP can assign a quantitative value to outcomes that are difficult to quantify in an objective optimization of stimulation parameters. BayesOpt efficiently finds optimal stimulation parameters and can be expanded to include additional motor signs and stimulation parameters. This is particularly important given the recent introduction of multi-segmented stimulation leads, and this approach may lead to improved patient outcomes.

## Data Availability

The datasets used and/or analyzed during the current study are available from the corresponding author on reasonable request.

## Abbreviations

DBS: Deep brain stimulation
PD: Parkinson’s disease
UPDRS: Unified Parkinson’s Disease Rating Scale
STN: Subthalamic nucleus
GPi: globus pallidus interna
AR: Akinetic-rigid
RoMaR: Robotic maniplandum rigidity
BayesOpt: Bayesian optimization
GP: Gaussian process
pGP: Probit Gaussian process
TEED: Total energy delivered.
